# Development of novel heminested PCR assays based on mitochondrial 16s rRNA gene for identification of seven pecora species

**DOI:** 10.1186/1471-2156-6-42

**Published:** 2005-08-11

**Authors:** Saurav Guha, VK Kashyap

**Affiliations:** 1 National DNA Analysis Centre, Central Forensic Science Laboratory, 30 Gorachand Road, Kolkata-700014, India

## Abstract

**Background:**

Characterization of molecular markers and the development of better assays for precise and rapid detection of wildlife species are always in demand. This study describes a set of seven novel heminested PCR assays using specific primers designed based on species-specific polymorphism at the mitochondrial 16S rRNA gene for identification of Blackbuck, Goral, Nilgai, Hog deer, Chital, Sambar and Thamin deer.

**Results:**

The designed heminested PCR assays are two consecutive amplifications of the mitochondrial 16S rRNA gene. In the first stage, ~550 bp region of the 16S rRNA gene was amplified by PCR using template DNA and universal primers. In the second stage, a species-specific internal region of the 16S rRNA gene was amplified by PCR using the amplicon of the first PCR along with one universal primer and another species-specific primer as the reverse or forward primer. The amplicon generated after two consecutive amplifications was highly unique to target species. These assays were successfully validated for sensitivity, specificity, and ruggedness under a wide range of conditions.

**Conclusion:**

The validation experiments confirm that the designed heminested PCR assays for identification of the seven species are highly specific, sensitive, reliable and provide a reproducible method allowing analysis of low copy number DNA recovered from decomposed or highly processed tissues. The assays for identification of other species could be devised by extrapolating the principle of designed heminested PCR.

## Background

Characterization of species-specific molecular markers and designing of species-specific assays for identification of wildlife species are essential to prevent illegal trade of parts and products for better conservation and management of endangered species. Illegal trade of skin, bone, horn, tail-hair, meat, antlers of Pecora family prevalent in India and have considerable trading value worldwide [1]. Laboratories often receive bio-specimens suspected to be of bovid and cervid origin. The protected members of Pecora family extensively hunted in India, e.g. Blackbuck, Goral, Nilgai, Chital, Thamin, Sambar, Hog deer, and Musk deer. *Antilope cervicapra *(Blackbuck), *Naemorhedus goral *(Goral) and *Boselaphus tragocamelus *(Nilgai) constitute three main Bovidae species endemic to India and adjoining countries. The Cervidae family also comprises of a number of species endemic to the Indian subcontinent e.g. *Cervus axis *(Chital), *Cervus unicolor *(Sambar), *Cervus eldi *(Thamin deer) and *Axis porcinus *(Hog deer). The density and distribution of these species have sharply declined due to loss of habitat through logging, livestock grazing and shift agriculture. All these species are prized for their meat, skin, horn, liver etc. and therefore extensively hunted [2]. All of these species are listed in IUCN (International Union for Conservation of Nature) and/or CITES (Convention on International Trade in Endangered Species of Wild Fauna and Flora) as vulnerable or threatened. In India they are listed in schedule I or III under Wildlife Protection Act, India (1972).

The conventional methods based upon the structural, electrophoretic and immunological characteristics of the species are often used in identification of skin, bone, horn, tail-hair, meat, antlers etc of poached animals [3,4]. However these methods are of limited use in species identification because of low stability and specificity of the markers. The biological materials forwarded for species identification are often of low quality. The analytical methods should be highly specific, sensitive, robust, reproducible and reliable. Presently DNA based techniques are extensively used in species identification [5-11]. Most DNA methods reported involve sequence analysis of mitochondrial DNA (mtDNA) [12-14]. The mitochondrial genome of vertebrates has been extensively used over nuclear DNA for resolving phylogenetic relationships at different evolutionary depth due to its unique properties, including presence of strictly orthologous genes, lack of recombination, and unique substitution rates [15]. These unique features along with high copy number of mitochondrial DNA per cell compared to nuclear DNA makes it a more powerful tool than nuclear DNA for identification of unknown biological materials [16-18]. Most of the designed species-specific identification methods are based upon mitochondrial Cytochrome b (Cytb) gene [19,20] due to the availability of well-documented universal primers for the gene [21]. The other genes also harbor species-specific sequences, are un-translated gene regions, e.g. rRNA genes, where base deletion and insertions can be tolerated without altering protein sequence, structure and function. Mitochondrial rRNA genes are also widely studied for evolutionary studies [22-24] and some recent studies also have proven the usefulness of unique polymorphisms present in mitochondrial rRNA genes in species identification [25-27]. However no report is available for mammalian species identification using species-specific polymorphism of mitochondrail rRNA genes.

DNA based methods employed in species identification comprised of species-specific PCR, RFLP-PCR, RAPD-PCR, SSCP, OLA etc are highly specific and sensitive but do not have inbuilt mechanism to assess DNA quality, quantity and presence of PCR inhibitor due to lack of a control step [7-10]. This control step ensures the quality of DNA amenable to amplify and detect the presence of any inhibitor in PCR reaction. The heminested PCR assay could integrate a independent control step with specific primer in two consecutive PCR reactions with improvement of sensitivity without impairing specificity [28].

In this study, we report a set of seven novel heminested PCR assays based on the amplification of a species-specific internal sequence region of mitochondrial 16s rRNA gene for identification of the species Blackbuck, Goral, Nilgai, Chital, Thamin, Sambar and Hog deer with their respective specific primers. To our knowledge, this is the first attempt to develop heminested PCR assay, exploring species unique polymorphism present in mitochondrial 16s rRNA gene for species identification.

## Results and discussion

The DNA based species identification methods often encounter low quantity and/or poor quality DNA template, and presence of PCR inhibitors. To examine these common yet serious problems, a independent control of DNA quality and quantity needs to be integrated in the identification assays. In this study we have designed and validated seven novel heminested mitochondrial 16s rRNA gene-targeted PCR assays for identification of seven species using designed species-specific primers listed in table 1. To design the species-specific primers we have screened the species unique sites or motifs in mitochondrial cytochrome b and 16s rRNA gene of thirty-seven species. Though level of polymorphism was high in cytochrome b gene compare to 16s rRNA gene, but species-specific motifs were higher in 16s rRNA gene among studied species [unpublished data. The species-specific unique sites at 16s rRNA gene were used to design the primers for identification of the studied species.

**Table 1:** Designed species-specific and selected universal oligonucleotide primers used in this study along with amplicon size.

Species	Primer name	Primer direction	Primer size (bp)	Primer sequence	Species – Specific amplicon size
All	16s Fwd	Forward	20	5'-CGCCTGTTTATCAAAAACAT-3'	
All	16s Rev	Reverse	20	5'- CTCCGGTTTGAACTCAGATC-3	~550 bp
*Antilope. Cervicapra*	BB16	Reverse	21	5'-ACCTAGTTATTCGCTATCAAG -3'	410 bp
*Naemorhedus. goral*	GL16	Reverse	29	5'-GGCAACTAGTTCAAAAAACCTAGTTATTT -3'	430 bp
*Boselaphus. tragocamelus*	NG16	Reverse	24	5'-AGTTATTCGCTATCTCACTAAACC -3'	408 bp
*Cervus. eldi*	TH16	Reverse	22	5'-CCTAGTTATTTGCTAACACACC -3'	400 bp
*Cervus. axis*	CH16	Reverse	25	5'-TATCCCAGAAGGACAGAATAATTAC -3'	240 bp
*Cervus. unicolor*	SM16	Forward	24	5'-GCTTTAACTACTTAGCCCAAAGAG -3'	350 bp
*Axis. porcinus*	HD16	Reverse	27	5'-CTCACAACAACAAAGGAATCGCTACTC -3'	300 bp

**Figure 1:**
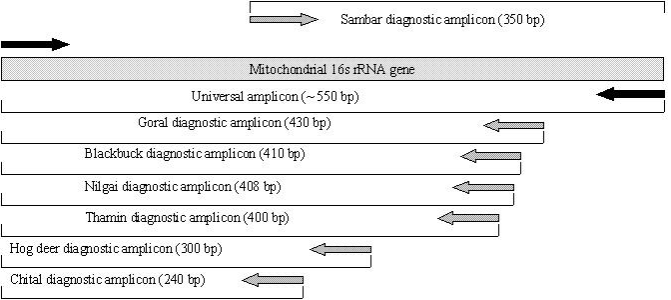
Schematic diagram of the mitochondrial 16s rRNA gene and species-specific amplicons with species – specific primers.

**Figure 2:**
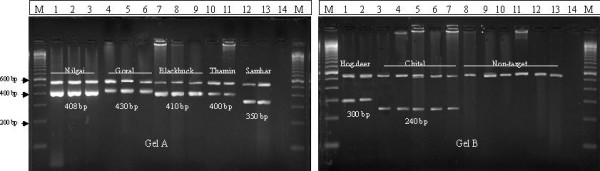
Species-specific profiles of designed heminested PCR assay. M indicates 100 bp ladders. Higher molecular weight amplicons were amplified for all the species with universal 16s rRNA primers. In 2^nd ^PCR only target species have generated species-species amplicons with specific primers. Lane 14 of both the gel contains negative controls.

In designed heminested PCR assays ~550 bp fragment were generated with universal outer primers at first cycle in all the studied species (Fig 2 and table 2). These results confirmed the quality of DNA amenable to amplification and nonexistence of any PCR inhibitor within the samples. It also enhanced the number of templates for next cycle of the assay. In the second PCR, using species-specific inner primers paired with another universal primer, all the individuals of a species generated species-specific amplicons (Fig 2 and table 2). The products of both amplifications for each individual were loaded together in to 3 % agarose gel for easy visualization. These species- specific amplicons are not present to any other non-target species having the 16s rRNA amplified region included in the study for testing the specificity of the method. The schematic diagram of species – specific amplicons with species – specific primers based on mitochondrial 16s rRNA gene are presented in fig 1. Though the diagnostic value of Blackbuck, Goral, Nilgai and Thamin deer specific amplicons are limited in 3 % agarose gel due to their almost equal fragment length but they are highly specific with specific primers.

**Table 2:** Results from species-specific heminested PCR assay with target and non target species for designed primers

Species	Common Name	DNA source	1^st ^PCR universal primer 16s rRNA gene	2^nd ^PCR BB 16	2^nd ^PCR GL 16	2^nd ^PCR NG 16	2^nd ^PCR TH 16	2^nd ^PCR CH 16	2^nd ^PCR SM 16	2^nd ^PCR HD 16
*Antilope cervicapra *a	Blackbuck	Tissue	+	+	-	-	-	-	-	-
*Antilope cervicapra *b	Blackbuck	Tissue	+	+	-	-	-	-	-	-
*Antilope cervicapra *c	Blackbuck	Blood stain	+	+	-	-	-	-	-	-
*Boselaphus tragocamelus *a	Nilgai	Tissue	+	-	-	+	-	-	-	-
*Boselaphus tragocamelus *b	Nilgai	Tissue	+	-	-	+	-	-	-	-
*Boselaphus tragocamelus *c	Nilgai	Tissue	+	-	-	+	-	-	-	-
*Naemorhedus goral *a	Goral	Blood stain	+	-	+	-	-	-	-	-
*Naemorhedus goral *b	Goral	Tissue	+	-	+	-	-	-	-	-
*Naemorhedus goral *c	Goral	Tissue	+	-	+	-	-	-	-	-
*Axis porcinus *a	Hog deer	Tissue	+	-	-	-	-	-	-	+
*Axis porcinus *b	Hog deer	Tissue	+	-	-	-	-	-	-	+
*Cervus axis *a	Chital	Blood stain	+	-	-	-	-	+	-	-
*Cervus axis *b	Chital	Tissue	+	-	-	-	-	+	-	-
*Cervus axis *c	Chital	Tissue	+	-	-	-	-	+	-	-
*Cervus axis *d	Chital	Tissue	+	-	-	-	-	+	-	-
*Cervus axis *e	Chital	Tissue	+	-	-	-	-	+	-	-
*Cervus eldi *a	Thamin	Tissue	+	-	-	-	+	-	-	-
*Cervus eldi *b	Thamin	Tissue	+	-	-	-	+	-	-	-
*Cervus unicolor *a	Sambar	Tissue	+	-	-	-	-	-	+	-
*Cervus unicolor *b	Sambar	Tissue	+	-	-	-	-	-	+	-
*Cervus duvaucelii*	Barasingha	Tissue	+	-	-	-	-	-	-	-
*Ovis aries*	Sheep	Tissue	+	-	-	-	-	-	-	-
*Bos tarus*	Cow	Tissue	+	-	-	-	-	-	-	-
*Homo sapiens*	Human	Blood	+	-	-	-	-	-	-	-
*Lepidochelys olevacea*	Turtle	Tissue	+	-	-	-	-	-	-	-
*Hemidactylus fluviviridis*	Lizard	Tissue	+	-	-	-	-	-	-	-

+ positive amplification, - negative amplification

**Figure 3:**
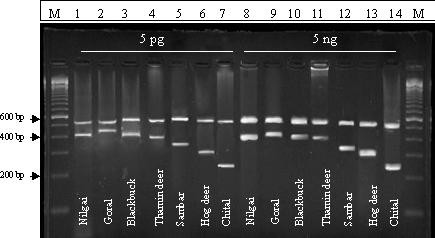
Species-specific profiles with 5 pg and 5 ng template DNA concentration of designed heminested PCR assay. M indicates 100 bp ladders. All the species have shown positive result at 1^st ^and 2^nd ^PCR.

The designed heminested PCR assays with species-specific primers successfully produced specific amplicon for the target species with consecutive dilution of DNA templates ranging from 5 ng to 5 pg (fig 3 and table 3). DNA extracted from various tissues generated identical result for all individual of a species using heminested PCR assay. To ensure the fidelity of the designed heminested PCR, a comparative experiment have been performed between heminested and normal PCR using DNA template isolated from a tissue of chital species. The normal PCR with 61°C annealing temperature using chital specific primer produced two nonspecific bands a long with the target band. On the otherhand, in heminested PCR assay using same annealing temperature only one single band of 240 bp size was produced at the second PCR reaction (fig 5). This experiment clearly reveals the higher fidelity of the designedheminested PCR compared to normal PCR used instudied species identification. The highly degraded DNAsample of a chital individual was also subjected for mitochondrial (16s rRNA) and nuclear (3' UTR of SON gene) gene amplification. The universal primers of 3' UTR of SON gene were not able to amplify the expected fragment of ~175 bp size. While, the universal primers of mitochondrial 16s rRNA gene successfully amplified a fragment of ~550 bp size. This result clearly indicates the usefulness of mitochondrial DNA in the designed assay over the nuclear DNA.

**Table 3:** Results of validation study of species-specific heminested PCR assays

Validation Parameters		No. of samples analysed	No. of specific primers analysed	No. of samples successfully typed
DNA concentration	5pg	20	7	20
	50 pg	20	7	20
	500 pg	20	7	20
	5 ng	20	7	20
Mixture analysis ^$^	Blackbuck + Nilgai + Goral + Hog deer + Chital + Thamin + Sambar	1 (mixture)	1^a^	1 (Blackbuck)
	Blackbuck + Nilgai + Goral + Hog deer + Chital + Thamin + Sambar	1 (mixture)	1^b^	1 (Nilgai)
	Blackbuck + Nilgai + Goral + Hog deer + Chital + Thamin + Sambar	1 (mixture)	1^c^	1 (Goral)
	Blackbuck + Nilgai + Goral + Hog deer + Chital + Thamin + Sambar	1 (mixture)	1^d^	1 (Hog deer)
	Blackbuck + Nilgai + Goral + Hog deer + Chital + Thamin + Sambar	1 (mixture)	1^e^	1 (Chital)
	Blackbuck + Nilgai + Goral + Hog deer + Chital + Thamin + Sambar	1 (mixture)	1^f^	1 (Thamin)
	Blackbuck + Nilgai + Goral + Hog deer + Chital + Thamin + Sambar	1 (mixture)	1^g^	1 (Sambar)
Chemical reagents	Soap	20	7	20
	0.1 N NaOH	20	7	20
	5% Acetic acid	20	7	20
	1 M NaCL	20	7	20
	Gasoline	20	7	20
Exposure to Heat	37°C, 1 week	7*	7	7
	37°C, 2 week	7*	7	7
	37°C, 3 week	7*	7	7
UV Irradiation (302 nm, 15 cm distance)	1 h	20	7	20
	8 h	20	7	20
	24 h	20	7	18

* One sample of each species were included for Exposure to Heat validation

^$ ^Each mixture comprised 50 pg of DNA of target species and 500 pg of DNA of each non-target species a – Blackbuck specific primer, b – Nilgai specific primer, c- Goral specific primer, d – Hog deer specific primer, e – Chital specific primer, f – Thamin specific primer, g – Sambar specific primer.

The designed heminested assays also successfully typed the target species from mixture of DNA originating from different species (table 3). The successful amplification of all the tissue samples treated with different chemical reagents confirms the strength of the assay (table 3). The tissue samples were also incubated at 37°C for different time period assuming the damp condition influencing the typing result. But all the samples were amplified successfully after this heat treatment (table 3). The results of analysis tissue samples subjected to UV irradiation indicate a minor influence of this factor on designed assay (table 3). These results confirm substantial stability and robustness of the assays against considerable influence of various chemical and physical factors.

The possibility of carry-over contamination is a common problem in nested or heminested PCR [26]. However these assays have been designed for highly species-specific primers that will prevent any non-specific amplification. Nevertheless, researchers need to setup separate pre- and post-PCR environments and take other precautions to avoid the risk of carry-over contamination.

## Conclusion

In conclusion, it is found that the designed heminested PCR assays based on unique species-specific polymorphism at mitochondrial 16s rRNA gene for identification of seven species are highly specific, simple and sensitive technique. Validation studies i.e. sensitivity of the designed assays compare to normal PCR, different DNA concentrations, species cross reactivity, specificity, and stability under various physical and chemical environment clearly reveal the applicability of the assays to less-than-optimal and highly degraded samples. Two consecutive PCRs and agarose gel visualization make the assays rapid as well as easy to interpret. The designed assays are more efficient and cost effective technology compared to other species identification tools, which do not have any inbuilt independent control of DNA quality and quantity. Because of highly species-specific primers, the designed heminested PCR assays provide reliable evidence for wildlife enforcement. These heminested PCR assays based on species-specific polymorphism in mitochondrial 16s rRNA gene can also be adapted for the identification of a wide range of wildlife species.

## Methods

### Samples and DNA isolation

DNA was extracted from various authenticated tissue samples of the unrelated individuals (N) of Blackbuck (N = 3), Goral (N = 3), Nilgai (N = 3), Thamin (N = 2), Chital (N = 5), Sambar (N = 2) and Hog deer (N = 2) by using standard phenol/chloroform procedure [29]. These samples of known animals species were provided by WII, Dehradun, India. DNA was concentrated and cleaned using Microcon 100 (Millipore) concentrators. Negative controls were included in every extraction. DNA was quantified using spectrophotometric method.

### Designing of primers

In heminested PCR the 16s rRNA gene based universal primers; 16s rRNA fwd and 16s rRNA rev [30] were chosen as outer primer and would amplify approximately ~550 bp amplicon. The species-specific internal primers were designed for blackbuck (BB16), goral (GL16), Nilgai (NG16), chital (CH16), thamin (TH16), sambar (SM16) and hog deer (HD16) (table 1). These specific primers were paired with the universal forward primer of first PCR, except for sambar where universal reverse primer used in the second PCR assay. These specific primers in conjunction with universal primers in heminested PCR generated specific amplicons; 410 bp (blackbuck), 430 bp (goral), 408 bp (Nilgai), 400 bp (Thamin), 240 bp (Chital), 350 bp (Sambar) and 300 bp (Hog deer) (table 1 and Fig 1).

### Primer specificity

The designed species-specific primers were subjected to BLAST search at NCBI GenBank database to rule out any false similarity with other species. These primers were also tested for amplification specificity using DNA extracted from a panel of non-target species belonging to different classes.

### Heminested PCR

DNA samples of individuals of studied species were subjected to heminested PCR assay with outer universal mitochondrial 16s rRNA gene primers at first PCR and with specific primers paired with one universal primer as reverse/ forward at second PCR. The first PCR reaction were carried out in a 25 μl reaction volume with 2 μl of DNA (10 ng – 30 ng/ μl) from tissue samples, 3 μl 10× PCR buffer, 2.5 μl of MgCl2 (15 mM), 2 μl of dNTP (2.5 mM each dNTP), 2 μl of both primers (10 pm/μl), 0.5 μl of Taq polymerase (5 U/μl), under the standardise condition; 94°C for 2 minute pre PCR denaturation, followed by 94°C for 30 seconds, 47°C for 30 seconds, 72°C for 45 seconds, for 30 cycles and a final extension at 72°C for 5 minutes. The PCR products of the first amplification with universal primers were diluted to ~1/20^th ^and 1 μl subjected to second PCR with universal 16s rRNA forward primer and Blackbuck, Goral, Nilgai, Thamin, Chital, and Hog deer specific primers as reverse at six different sets and universal 16s rRNA reverse primer and Sambar specific primer in a single set. The second PCR cycles were carried out almost in identical conditions to the first PCR cycle except the species – specific primer annealing temperature and final MgCl_2 _concentration. The annealing temperature and final MgCl_2 _concentration for Blackbuck and Goral specific primers is 56°C and 0.9 mM, Nilgai specific primer is 54°C and 1.5 mM, Chital and Hogdeer specific primer is 61°C and 1.0 mM, Thamin deer is 58°C and 1.0 mM and Sambar is 62°C and 1.0 mM. The amplicons of both the PCR were loaded together for an individual of a species in a single lane and visualized in 3% agarose gel with 100 bp ladder, a negative control and with other non-target species performed the same assay (Fig 2). The results of all the heminested PCR assays are presented at table 2.

### Validation study

Sensitivity of these assays were assessed by analysis of consecutive dilution of total DNA extracted from different tissues (muscle, liver, heart, kidney, skin) and blood stains of unrelated individuals of seven species. The DNA dilutions were prepared in a range of 5 pg to 5 ng and subjected to PCR assay with designed specific primers using stringent conditions described above (table 3, fig 3).

Fidelity of the designed heminested PCR assays compare to normal PCR assay was also evaluated by using the DNA template isolated from tissue sample of chital species along with chital specific primer.

To check the suitability of using mitochondrial DNA over nuclear DNA we have extracted DNA from a highly degraded and mutilated chital tissue sample. No high molecular DNA was found and the DNA aliquot was subjected to PCR with mitochondrial 16s rRNA gene universal primers and 3' UTR of the SON gene [31] universal primers (SON Fwd primer 5' – ACATAGCATATGAATACC – 3' and SON Rev primer 5' – GTCTATCTAGGTGTAGCTGA – 3').

Mixture analysis was also performed with all the designed species-specific primers to evaluate the sensitivity of detection from mixture of DNA samples of more than one origin. Seven types of DNA mixtures were prepared and subjected to heminested PCR assays consecutively using seven designed primers (table 3). Each mixture was prepared by aliquoting 50 pg of DNA of target species and 500 pg of DNA of non-target species.

#### Exposure of samples to different factors

Biospecimens of known species were exposed to different chemical reagents (gasoline, soap, 0.1 N NaOH, 5% Acetic acid, 1 M NaCl) and analyzed after 5 days of storage in ambient temperature. Small pieces of tissue samples were also placed in dishes floating in a water bath settled at 37°C for periods of 1, 2 and 3 week. After the exposure samples were kept frozen in -80°C until extraction. Samples were also treated by UV irradiation (302 nm) from a distance of 15 cm for 1 h, 8 h and 24 h (table 3).

## Authors' contributions

SG carried out the experiments of standardisation and validation of heminested PCR assays and significantly contributed in preparation of manuscript.

VKK conceived, designing of experiments, and contributed in preparation of manuscript.
